# Dynamic reprogramming of chromatin: paradigmatic palimpsests and HES factors

**DOI:** 10.3389/fgene.2015.00029

**Published:** 2015-02-10

**Authors:** Kurtulus Kok, David N. Arnosti

**Affiliations:** ^1^Genetics Program, Michigan State University, East Lansing, MI, USA; ^2^Department of Biochemistry and Molecular Biology, Michigan State University, East Lansing, MI, USA

**Keywords:** HES, hairy, oscillatory gene expression, repression, chromatin

## Abstract

Temporal and spatial control of transcription in development is dictated to a great extent by transcriptional repressors. Some repressor complexes, such as Polycomp-group proteins, induce relatively long-term non-permissive states, whereas others such as hairy/enhancer of split (HES) family repressors are linked to dynamically modulated chromatin states associated with cycling expression of target genes. The mode of action and specificity of repressors involved in mediating this latter form of epigenetic control are unknown. Oscillating expression of HES repressors controlled by signaling pathways such as Notch suggests that the entire ensemble of HES–associated co-repressors and histone modifying complexes readily cycle on and off genes. Dynamic interactions between these factors and chromatin seem to be crucial in maintaining multipotency of progenitor cells, but the significance of such interactions in more differentiated cells is less well understood. We discuss here how genome-wide analyses and real-time gene expression measurements of HES regulated genes can help decipher the detailed mechanisms and biological importance of highly dynamic transcriptional switching mediated by epigenetic changes.

## INTRODUCTION

**FIGURE 1:**
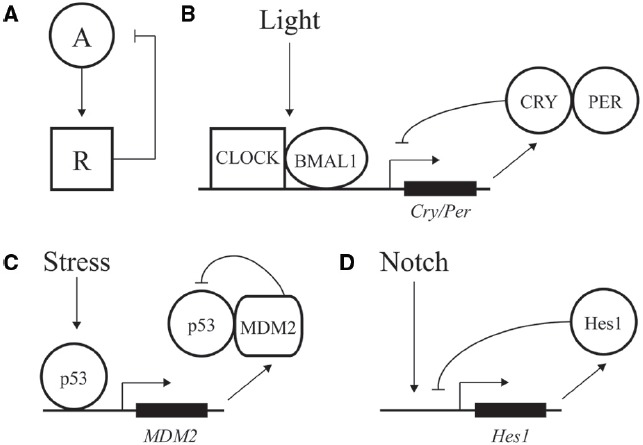
**Negative feedback loops at the core of transcriptional oscillators. (A)** Diagram of a simple negative feedback loop for oscillatory behavior. An activator “A” increases activity of a repressor “R,” which in turn decreases activity of the activator. **(B)** Major factors driving daily oscillations of the circadian clock, whereby CLOCK/BMAL1 drive expression of the inhibitory factors CRY/PER. **(C)** Stress and DNA damage activation of the p53 pathway, whereby 5–9 h. ultradian oscillations in p53 activity drive expression of p53 inhibitor MDM2. **(D)** Hes1 expression is driven by Notch signaling and feedback inhibited by Hes1, with an oscillation of ∼2–3 h.

Dynamic cellular processes in biological systems require modulated and adaptable responses at the level of gene expression. Variations in the internal and external environment provoke short-and long-term changes in gene expression, which help maintain cellular physiology; these controls are also a fundamental point of evolutionary changes (López-Maury et al., 2008). Some variability in output of gene regulatory networks (GRN) is an inescapable consequence of molecular noise, including stochastic switching of promoter activity or “bursts.” Such random fluctuations can be easily propagated to downstream genes or buffered out, and may play significant roles in physiological regulation, differentiation, adaptation and evolution (Eldar and Elowitz, 2010). In addition to the impact of stochastic molecular processes on gene expression, organisms from bacteria to animals have evolved a wide variety of specialized oscillatory gene expression mechanisms to respond to predictable and unpredictable environmental fluctuations and effect developmental programs (Young and Kay, 2001; Paszek et al., 2010). The levels of mechanistic complexity vary among oscillatory systems, but they share common regulatory principles, including negative feedback loops (Figure 1A). These core features were successfully used to design simple synthetic oscillatory networks that accurately predict the dynamic behavior of biological systems, which are generally more complex and feature robustness to genetic and environmental influences (Elowitz and Leibler, 2000; Cookson et al., 2009; Tigges et al., 2009).

## DESIGN AND FUNCTION OF OSCILLATING GENE NETWORKS

A classic example of oscillatory transcriptional regulation is the ability of the circadian clock to adjust output of many genes in preparation for predictable daily changes in light, food, and temperature (Bell-Pedersen et al., 2005). Although regulation is highly complex, the core of the vertebrate molecular clock is based on transcriptional activation of genes under control of the CLOCK and BMAL1 activators. These factors drive expression of many genes during the day, including the PER and CRY repressors, which feedback inhibit and block CLOCK/BMAL1 action during the nighttime (Figure 1B; Ko and Takahashi, 2006; Baggs and Hogenesch, 2010). Repression is relieved by phosphorylation, ubiquitination, and degradation of PER and CRY, leading to a feedback loop with a period of ∼24 h (Busino et al., 2007).

Genome-wide studies have revealed associated rhythmic changes of histone marks corresponding to oscillatory expression of thousands of genes coordinating biological cycles through a complex regulatory network (Feng et al., 2011; Koike et al., 2012). A recent study from the Takahashi laboratory provided a comprehensive overview of chromatin-associated dynamics of circadian cycling in the murine liver. Using time-dependent ChIP-seq analysis of transcription factors (BMAL1, CLOCK, NPAS2, PER1, PER2, CRY1, CRY2, p300, and CBP), RNA Pol II, and histone marks (H3K9Ac, H3K27Ac, H3K4me1, H3K4me3, H3K36me3, and H3K79me2), the authors identified three phases in the circadian clock corresponding to genes in a transcriptionally poised, activated, and repressed states (Koike et al., 2012).

In addition to predictable daily cycles, cells need to respond to rapid changes and variations during development and growth. Ultradian oscillations often feature a time period of minutes to hours and are triggered by intrinsic and environmental signals. One of the best described such instances is represented by the p53 pathway; this transcription factor can display dynamic behavior in response to DNA damage and other cellular stress to protect cells against malignant transformation (Batchelor et al., 2011). p53 expression is regulated by a negative feedback loop. The MDM2 regulator normally keeps p53 activity at low levels by binding to the factor’s DNA binding domain, inducing a change in subcellular localization from the nucleus to the cytoplasm, and inducing ubiquitylation for eventual degradation of p53 (Wu et al., 1993; Haupt et al., 1997). After DNA damage, the p53 protein is phosphorylated, preventing the interaction of MDM2 with p53 and resulting in activation of p53 (Kruse and Gu, 2009). p53 transcriptionally activates expression of many genes including *MDM2*, resulting in a time-delay feedback inhibition that can exhibit oscillations of both p53 and Mdm2 (Figure 1C; Lev Bar-Or et al., 2000; Lahav et al., 2004; Bose and Ghosh, 2007). Depending on the dynamic control of p53, different cellular responses can be elicited. Cells can undergo a transient cell cycle arrest and recover from the DNA damage (Purvis et al., 2012). In addition to transient responses, the p53 pathway also triggers terminal fates such as apoptosis and senescence. In contrast to oscillatory output, sustained p53 expression affects the expression of a different set of genes, leading to senescence (Purvis et al., 2012). Therefore, depending on the dynamics of the input, distinct chromatin and regulatory changes can be imparted on a gene network to transmit information and alter cellular fate.

Oscillations are also seen in differentiation and embryonic development. One of the best-studied examples involves the transcriptional repressor Hes1 that controls the differentiation of neurons and formation of somite segments in the vertebrate hindbrain (Figure 1D; Kageyama et al., 2007; Koike et al., 2012). Hes1 belongs to the conserved family of hairy/enhancer of split (HES) transcriptional repressors that recruit common co-repressors of the Groucho/TLE family (Davis and Turner, 2001; Aloia et al., 2013). The eponymous Drosophila Hairy repressor functions as a so-called long-range repressor that remodels large blocks of chromatin upon transcriptional repression. Hairy mediates wide-spread and coupled loss of active histone marks H4Ac, H3K27Ac, H3K4me1, and H3K4me3 on many embryonic genes (Li and Arnosti, 2011; Kok et al., in review). Furthermore, Hairy represses its own transcription by removing these active marks, consistent with the previously observed autoregulatory mechanism of related mammalian HES proteins (Kageyama et al., 2007).

A conserved feature of regulatory pathways involving HES proteins is the role of Notch signaling. Upon ligand binding, Notch is cleaved and released from the plasma membrane to translocate to the nucleus, where it associates with and activates the *Hes1* promoter. Hes1 protein negatively regulates its own promoter, establishing a feedback loop (Fischer and Gessler, 2007). This feedback loop can induce oscillations in Hes1 protein levels (Kageyama et al., 2007). Periodic temporal expression of Hes1 plays a crucial role in formation of somites, which give rise to the vertebrae, ribs, skeletal muscles and dermis (Aulehla and Herrmann, 2004). These segments are formed from the anterior region of the presomitic mesoderm (PSM) by periodic Notch signals. Notch coordinates Hes1 oscillations, which progress from the posterior to anterior region of the PSM. One wave of expression of this so-called segmentation clock lasts 2 h, marking the boundary for a new somite that forms at the end of the embryo (Pourquié, 2003). In this setting, temporal oscillations are converted into a spatial pattern of somite boundaries. A large number of genes involved in cell signaling are periodically expressed during this segmentation process in mouse (Dequéant et al., 2006). Comparison of the mouse, chicken and zebrafish PSM oscillatory transcriptomes revealed networks of 40–100 conserved cycling genes that are activated downstream of the Notch, Fibroblast Growth Factor and Wnt pathways (Krol et al., 2011). Thus, the segmentation clock is controlled by conserved multiple signaling pathways. The common oscillatory genes in all vertebrates include at least one member of the Hes/Her family. However, the identity of cyclic genes varies from species to species as well, indicating evolutionary plasticity of the segmentation networks (Krol et al., 2011).

In contrast to the fate-determining effects of Hes1 oscillations in the PSM, cyclic behavior of Hes1 in neuronal progenitor cells (NPC) is associated with stabilization of the undifferentiated phenotype. In these cells, Hes1 mRNA, protein, and activity oscillate with a 2 h period (Hirata et al., 2002). Hes1 represses transcription of proneural transcription factors such as *Ascl1*, inducing oscillations in levels of that factor. Interestingly, self-renewal of NPCs and their eventual proper differentiation is achieved only when Hes1 and downstream genes are periodically expressed (Imayoshi and Kageyama, 2014). Sustained expression of Hes1 constitutively in NPCs represses proneural genes, blocking proliferation and inducing quiescence (Baek et al., 2006). This observation indicates that active division of NPCs is dependent on the oscillatory expression of fate determination factors. Neuronal fate choice is determined by sustained expression of Ascl1 after cell division. During differentiation, Hes1 oscillations cease as Notch inputs diminish, leading to upregulation of Ascl1 (Imayoshi et al., 2013). Using a light-activatable system, the impact of oscillating and sustained expression of Ascl1 on proliferation and differentiation of NPCs was tested. A 3 h periodic expression of Ascl1 supported proliferation of NPCs, whereas sustained expression resulted in differentiation (Imayoshi et al., 2013). Similar roles for Hes1 oscillation has been observed in embryonic stem cells (Kobayashi et al., 2009).

The types of chromatin dynamics occurring on genes entrained under the circadian clock system have not been well documented for oscillations involving ultradian factors such as HES proteins and other bHLH transcription factors. However, a recent study suggested that the Ascl1 bHLH factor, which shows oscillatory expression complementary to that of Hes1 in neuronal progenitors, is critical for formation of open chromatin during reprogramming through its activities as a pioneer factor on enhancers (Wapinski et al., 2013). Less is known about the chromatin modifying properties of Hes1 itself, however, the homologous Drosophila protein Hairy has a direct role in chromatin modification, and this protein impacts the chromatin state of hundreds of loci on a genome-wide scale (Li and Arnosti, 2011; Kok et al., in review). As HES transcription factors share common structural features, including DNA binding and effector domains, as well as conserved developmental roles, the biochemical properties are likely to be similar.

How general are the dynamic chromatin responses associated with activation and repression of genes such as those targeted by HES factors? The time-delays associated with activating or repressing promoters are a function of dynamics of protein complexes. Even in steady-state situations, transcription factors are observed to continuously associate and dissociate with target loci, a feature not revealed by ChIP experiments but that is demonstrated by direct imaging as well as *in vitro* approaches (Voss and Hager, 2014). However, as observed for the prolactin promoter, stochastic chromatin processes can render promoters refractory to stimulation. Such refractory periods would block transmission of dynamic signals (Harper et al., 2011). Indeed, high-resolution temporal measurement of mRNA of many mammalian genes from single cells reveals that distinct regulatory regions confer gene-specific switching rates with different refractory periods (Suter et al., 2011). Such differences may cause differential oscillation of genes in response to stimuli. Fine time-scale analysis of global gene expression triggered by the inflammatory cytokine TNF showed oscillations in > 5000 genes that are involved in multiple pathways, with different genes oscillating either very rapidly or after a lag phase (Sun et al., 2008). Cyclic interaction of transcription factors with promoters can extend from seconds for bursting promoters to minutes for developmental oscillators to hours for circadian clocks. A single promoter may experience both fast (2 min) and slow (40 min) periodic binding of a single transcription factor, as with Ace1 occupancy of the yeast *CUP1* promoter (Karpova et al., 2008). The authors suggest that fast cycling is responsible for the initial period of gene expression, while slow cycling represents the fine-tuning of expression levels associated with slow-period oscillating nucleosome occupancy. A short-period ultradian cycling has also been described for the estrogen receptor, involving periodic binding and assembly of chromatin complexes in mammalian cells, however, recent high-resolution studies of RNA polymerase activity have not supported this picture (Hah et al., 2011; Voss and Hager, 2014).

In development, oscillatory circuits affect not only specific networks of genes relating to patterning, as described for Hes1, but also can include many synchronized genes not linked to circadian control. Large-scale transcriptome analysis in *C. elegans* larvae revealed robust ∼8 h cycling of thousands of genes, which may be related to developmental processes such as molting (Hendriks et al., 2014). In contrast to the simple synthetic biology circuits tested in bacteria, such large-scale oscillatory behavior likely involves more components than a single negative feedback loop (Sun et al., 2008). The coordinated expression of many genes in these systems indicates that persistent chromatin changes are not likely to prevent genome-wide oscillatory coordination, thus the dynamic chromatin changes found for HES factors are likely to be representative of many regulatory mechanisms.

## OSCILLATORY BEHAVIOR AND CHROMATIN DYNAMICS

**FIGURE 2:**
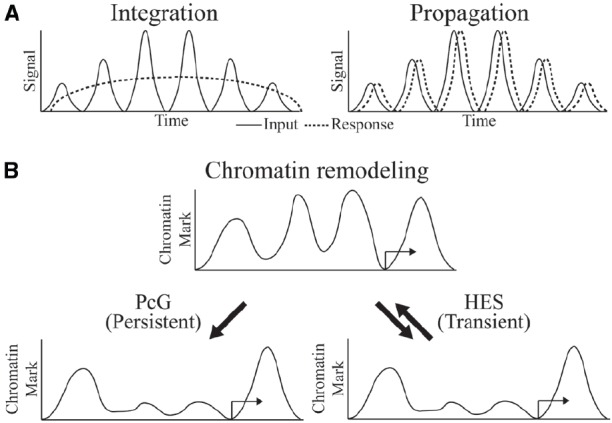
**Oscillator inputs and possible outcomes. (A)** The oscillating input signal can be integrated or propagated to generate a sustained or dynamic response, respectively. Time on the x-axis might be minutes to hours. **(B)** Outcome at the chromatin level might be persistent and long-term in integrated response by PcG regulation or transient and reversible in propagated response by HES regulation.

The biochemical mechanisms by which transcriptional oscillations can be induced are in many cases better understood than the physiological significance of such dynamics. In the case of circadian regulated genes, adaptation to predictable environmental changes, such as food availability, temperature or light, is a clear driver of such dynamics. In development, the dynamic readout of HES activity represents a morphological pattern generator. In other cases, it is not clear whether the cycling is a necessary feature of the system, or tolerated as an also-acceptable form of control that may or may not have superior regulatory properties. Arguing against a view that cycling occurs by chance is the likelihood that randomly propagated oscillations though a multi-level network should eventually cancel out, thus it is likely that there is selection for coordinated responses at some level. Depending on the nature of downstream targets, cycles of transcriptional output may be “integrated” to a steady-state approximation of the average level of signaling, or it may be “propagated,” if dynamics of the downstream gene expression is as fast as the cycling signal (Hoffmann, 2002; Figure 2A).

Oscillatory behavior may be eventually damped by several layers of a gene regulatory cascade. For example, in the case of cyclical expression of Hes1, expression of several downstream targets also alternates, but the overall undifferentiated state of the cell—represented by the global activity or inactivity of many genes—stays constant, indicating that at least at a larger scale, such oscillatory behavior is subsumed into a stable phenotype. Alternatively, the oscillatory action at one level of a GRN may better ensure that a particular level of expression within a critical range is maintained, rather like a singer who uses vibrato to hold a particularly difficult note (Imayoshi et al., 2013). At the same time, the interlocking feedback loops that permit oscillation also provide the control points that can be shifted to move a cell into a different gene regulatory, and eventually differentiated state. These arguments are attractive in pointing out possible adaptive features of oscillatory regulation, however, testing the null hypothesis is difficult. It may be that just as transcriptional “bursting” is an inevitable consequence of micro-scale chromatin movements, longer period, regular transcriptional oscillations may be system properties that arise as a secondary consequence of core properties of the system, such as robustness. Alternatively, or in addition, many oscillations that are observed are consequences of a few key dynamic drivers that must show periodic changes; the ancillary downstream changes may not important for natural selection acting on gene expression (Paszek et al., 2010; Cheong and Levchenko, 2010).

What is known about the required chromatin dynamics that are associated with oscillatory gene regulation? Circadian regulated genes exhibit cyclical chromatin responses that reset every day (Koike et al., 2012). In the developmental settings for Hairy and HES protein activity, the targets of these proteins are often active only transiently, implying very dynamic chromatin responses. For instance, the activators of *ftz*, a gene that is repressed by Hairy, are present on the genome for only minutes during early embryogenesis, and repressive countermeasures would be required only for a similarly brief time. Indeed, we find that in cases of artificial induction of Hairy, dramatic chromatin deacetylations are quickly reversed as soon as Hairy levels drop, indicating that the repressor is working against a background of cellular chromatin modifying activities that quickly restore a landscape to the status quo ante (K. Kok, unpublished observations). Hes1 action, although not studied at the chromatin level, must similarly be transient in terms of perdurance, as downstream transcriptional targets quickly follow changes in the levels of Hes1 over a period of hours. Thus, in general, HES protein directed alterations to genome-wide chromatin states may be very transient (Figure 2B). In some regulatory circuits, we do know that chromatin states are locked in, preserving a particular epigenetic mark through multiple mitoses—these markers involve Polycomb complexes in Drosophila and higher metazoans, as well as DNA methylation signals in vertebrates. Significantly, both of these systems can be deployed in alternate modes, so that in some instances DNA methylation and Polycomb-regulated effects are transient (Aloia et al., 2013). Are global chromatin modifications just reflections of gene regulatory effects rather than drivers of the system? To what extent are these chromatin changes important for setting the boundary conditions for oscillatory gene responses? Systems and synthetic biology approaches will converge with developmental gene regulation to deliver answers to these intriguing questions.

### Conflict of Interest Statement

The authors declare that the research was conducted in the absence of any commercial or financial relationships that could be construed as a potential conflict of interest.
